# Hematopoietic Cell Kinase (HCK) Is a Player of the Crosstalk Between Hematopoietic Cells and Bone Marrow Niche Through CXCL12/CXCR4 Axis

**DOI:** 10.3389/fcell.2021.634044

**Published:** 2021-03-25

**Authors:** Fernanda Marconi Roversi, Maura Lima Pereira Bueno, Fernando Viera Pericole, Sara Teresinha Olalla Saad

**Affiliations:** Hematology and Transfusion Medicine Center—University of Campinas/Hemocentro-Unicamp, Instituto Nacional de Ciência e Tecnologia do Sangue, Campinas, Brazil

**Keywords:** acute myeloid leukemia, cancer therapeutic target, CXCL12/CXCR4 axis, hematopoietic cell kinase, PI3K/MAPK pathway, crosstalk, bone marrow—pathology

## Abstract

The crosstalk between hematopoietic stem/progenitor cells (HSC), both normal and leukemic, and their neighboring bone marrow (BM) microenvironment (niche) creates a reciprocal dependency, a master regulator of biological process, and chemotherapy resistance. In acute myeloid leukemia (AML), leukemic stem/progenitor cells (LSC) anchored in the protective BM microenvironment, reprogram and transform this niche into a leukemia-supporting and chemoprotective environment. One most important player involved in this crosstalk are CXCL12, produced by the BM mesenchymal stromal cells, and its receptor CXCR4, present onto HSC. The downstream molecular mechanisms involved in CXCL12/CXCR4 axis have many targets, including the Src family members of non-receptor tyrosine kinase (SFK). We herein study the role of one SFK member, the Hematopoietic Cell Kinase (HCK), in CXCL12/CXCR4 pathway and its contribution to the AML pathogenesis. We verified that the inhibition of HCK severely impaired CXCL12-induced migration of leukemic cell lines and CD34 positive cells from AML patients bone marrow, through a disruption of the activation of CXCL12/CXCR4/PI3K/AKT and CXCL12/CXCR4/MAPK/ERK signaling, and by a decreased cytoskeleton dynamic through a lower rate of actin polymerization. We provide new insights into the key role of HCK in conferring a migratory advantage to leukemic cells thought CXCL12/CXCR4 axis. HCK represents an important protein of the main pathway involved in the crosstalk between HSC, and their surrounding milieu. Thus, HCK inhibition could represent a novel approach for the treatment of the acute myeloid leukemia.

## Introduction

Acute myeloid leukemia (AML), the most common acute leukemia in older adults (60 years of age or over), is characterized by an uncontrolled myeloid cell expansion, resulting in an impaired capacity to differentiate into fully mature cells that could culminate in bone marrow failure (BM) (Siveen et al., 2017; El-Jawahri et al., 2019). During normal hematopoietic stem/progenitor cell (HSC) differentiation, some genetic mutations can occur, leading to a clonal evolution and leukemic stem/progenitor cell (LSC) formation. These malignant cells are able to use the neighboring bone marrow microenvironment (niche) for their expansion, self-renewal, and survival, without being recognized, thus inducing leukemic progression. Furthermore, this crosstalk between LSC and the BM niche is also an important mediator of chemotherapy resistance, and disease relapse (Ladikou et al., 2020).

The transmembrane G protein-coupled C-X-C chemokine receptor type 4 (CXCR4) and its chemokine ligand CXCL12 (stromal derived factor-1 alpha; also known as SDF-1) are important players involved in the crosstalk between HSC or LSC and the BM microenvironment (Shafat et al., 2017). CXCL12 is produced by the bone marrow mesenchymal stromal cells (BM-MSC) and binds to the CXCR4 present in the HSC or LSC, resulting in the modulation of diverse processes, such as chemotaxis, cell survival, cell growth, cell adhesion, and gene transcription (de Lourdes Perim et al., 2015). Furthermore, due to the higher CXCL12 secretion by AML BM-MSC or CXCR4 superexpression in LSC, the leukemic cells have a greater ability to modulate CXCL12/CXCR4 axis facilitating their trafficking and homing into the protective BM microenvironment, maintaining their quiescence and protected from chemotherapeutic compounds (Wang and Zhong, 2018). Notably, high *CXCR4* gene expression or protein activation into AML CD34 positive blasts is known to be associated with poor prognosis of AML patients (Du et al., 2019).

The CXCL12/CXCR4 axis activates many downstream targets in HSC or LSC, including Src family kinase (SFK) members (Amata et al., 2014). Deregulation of SFK is frequently observed in diverse human cancer and its upregulation has been associated with poor clinical prognosis and tumor recurrence (Dubois et al., 2015). Interestingly, our group identified high expression of a SFK member, the hematopoietic cell kinase (HCK), in primary CD34 positive hematopoietic cells isolated from myelodysplastic syndrome (MDS) patients bone marrow (Roversi et al., 2017) and from *de novo* AML patients bone marrow (Baratti et al., 2010), a clonal hematopoietic stem cell disorder with a risk of 30% to progress toward AML (Chen et al., 2019).

In light of these facts, we explored the role of HCK in the CXCL12/CXCR4 axis as well as the possible contribution of HCK in AML pathogenesis. We used a targeted siRNA lentivirus or a specific pharmacological inhibitor to downregulate *HCK* gene expression and/or HCK protein activity and investigated their involvement in chemotaxis, actin polymerization, and related signaling pathways. Notably, we observed that HCK gene and protein depletion was able to abrogate the increased chemotactic migratory ability stimulated by CXCL12. Our data also showed that actin polymerization after HCK inhibition did not exhibit a characteristic induction peak of actin polymerization after CXCL12-chemotaxis stimulus, in presence or absence of CXCR4 antagonist, which probably indicated that HCK protein is located downstream of CXCR4. We also observed a decrease in phosphorylation of AKT and ERK proteins after HCK downregulation, even following a CXCL12 stimulus. Herein, we provide new insights to the role of HCK as an intermediate target of CXCL12/CXCR4 and PI3K/AKT or MAPK/ERK signaling pathway, representing an important protein of the crosstalk between HSC, and their surrounding milieu. HCK probably acts as an oncogene in AML, which supports the exploration of pharmacological HCK inhibitors in future AML preclinical and clinical studies.

## Materials and Methods

### Cell Lines and Cultures

The human myeloid leukemia cell lines U937 and KG1a were obtained from ATCC (Philadelphia, PA, United States) and were cultured in RPMI-1640 supplemented with 10% Fetal Bovine Serum (FBS), L-glutamine, amphotericin B, and 1% penicillin-streptomycin maintained at 37°C and 5% CO_2_. Cells were harvested during the log phase of growth.

Mononuclear cells obtained from bone marrows of AML patients (*n* = 5) were isolated using Ficoll-Hypaque. After red blood cell lysis, primary CD34^+^ BM cells were purified by magnetic bead separation using the human CD34 MicroBead kit and the AutoMACS Pro separator (Miltenyi Biotec). Purity of the CD34^+^ fraction was assessed by flow cytometry with anti-CD34-phycoerythrin (PE) antibody (BD Biosciences). CD34^+^ fractions showing purity greater than 90% were used. Cells were cultured in StemSpan media (Stem Cell Technologies) supplemented with 20% FBS, cytokines, including SCF, FLT-3L, TPO (all at 300 ng/mL), IL-3 (180 ng/mL), IL-6 (30 ng/mL), L-glutamine, amphotericin B, and 1% penicillin-streptomycin, maintained at 37°C with 5% CO_2_.

### Patients

All AML patients included in this study were untreated at the time of sample collection and followed at the outpatient clinic of Hematology and Transfusion Medicine Center of the University of Campinas. The study was approved by the Human Ethics Committee of the University (CAAE 1110.0.146.000-11), in accordance with the Code of Ethics of the World Medical Association (Declaration of Helsinki). All patients signed informed consent forms.

### Reagent Chemicals

A selective HCK inhibitor, iHCK-37 (ASN05260065; ASINEX company), was diluted in dimethylsulfoxide (Me_2_SO_4_; DMSO) to a 100 mM stock solution. AMD3100 (Sigma-Aldrich) was diluted in water to a 100 mM stock solution. Recombinant Human SDF-1α (CXCL12, PeproTech) was diluted in PBS buffer to a 100 ng/μL stock solution.

### Lentiviral Transduction by RNA Interference

Cell lines were transduced with control sequence shRNA (sc-108080, Santa Cruz and BlockIT shRNA Vectors, Invitrogen) or HCK shRNA (sc-35536-V, Santa Cruz, and BlockIT shRNA Vectors, Invitrogen) by spinoculation in the presence of polybrene (6 μg/mL, Invitrogen). The transduced cells were selected for 10–15 days using puromycin (Santa Cruz Biotechnology). Puromycin resistant cells were expanded and analyzed for functional assays.

CD34^+^ cells isolated from the bone marrow of AML patients were loaded on a RetroNectin-coated plate (Takara). After 24 h in culture, the CD34^+^ cells were transduced with lentivirus expressing shRNA against HCK (shHCK) or an appropriate control sequence (shControl) in the presence of SCF, FLT-3L, TPO (all at 300 ng/mL, PeproTech), IL-3 (180 ng/mL, PeproTech), IL-6 (30 ng/mL, PeproTech), and protamine sulfate (4 μg/mL, Sigma-Aldrich). Cells were sorted for green fluorescent protein (GFP) using a FACS Aria III (Becton Dickinson).

### Quantitative Polymerase Chain Reaction (qRT-PCR)

Total RNA was extracted from leukemia and primary cells, using RNeasy Micro Kit (Qiagen). cDNA was produced using RevertAid H Minus First Strand cDNA Synthesis Kit (Thermo Fisher Scientific). qRT−PCR was performed with the reagent SYBR Green Master Mix PCR (Thermo Fisher Scientific) and analyzed by ABI 7500 Sequence Detection System (Applied−Biosystem). The relative quantification of gene expression values were calculated using the equation 2^–ΔΔ*CT*^ with the housekeeping genes *hypoxanthine guanine phosphoribosyltransferase 1* (*HPRT1*), *beta actin* (*ACTB*), and *glyceraldehyde−3−phosphate desidrogenase* (*GAPD*H) (Livak and Schmittgen, 2001) and each data have been normalized to the values of the control. A control was used for each primer pair. Amplification specificity was verified using a dissociation curve at the end of each run. Each experiment was performed in triplicate on the same plate for each sample.

### Chemotaxis Assay

Transduced leukemic cell lines (KG1a and U937), and transduced CD34^+^ cells from AML patients as well as leukemic cell lines (KG1a and U937) pretreated or not with iHCK-37 (48 h of stimulation) were tested for chemotaxis in Transwell-based assays Chemotaxis (6.5 mm diameter, 8 μm pore size; Corning). For treatment with antagonist of CXCR4, cells were incubated with 1.25 μg/mL of AMD3100 (Sigma-Aldrich) for 1 h and then submitted to transwell migration assay. Polycarbonate membranes were incubated with poly-L-lysine (1mg/mL) for 1 h at 37°C and then washed twice with PBS. Briefly, cells in each condition were washed twice with RPMI containing 0.1% BSA, then seeded at a density of 5 × 10^5^ cells into the upper chamber of the transwell. The lower chamber was filled with RPMI containing 0.5% bovine serum albumin (BSA; negative control), RPMI containing 10% FBS (positive control) and RPMI containing 0.5% BSA with CXCL12 (100 ng/mL, Peprotech) (Melo et al., 2014). Following 6 or 24 h of incubation at 37°C, cells that migrated into the lower chamber were collected, centrifuged, resuspended in phosphate buffered saline (PBS) buffer and counted. The number of migrated cells was expressed as a percentage of the input, i.e., the cells number applied directly to the lower compartment in parallel wells. The migration of cells was normalized to 100% ± standard deviation (SD) of triplicates (Favaro et al., 2013).

### Actin Polymerization Assay

F-actin polymerization was analyzed in transduced or treated leukemic cell lines (KG1a and U937) using AlexaFluor 633-labeled phalloidin (A22284, Invitrogen) before and after CXCL12 stimulation. Treatment with AMD3100 was performed as above and then cells were submitted to CXCL12 stimulus. Briefly, cells were stimulated with CXCL12 (300 ng/mL; PeproTech) in serum-free RPMI at 37°C for 30 and 120 s. The reaction was stopped by adding 3 volumes of 3.7% paraformaldehyde at room temperature for 10 min, then washed and permeabilized on ice. Cells were stained with 633–phalloidin (2 mg/mL). Flow cytometric data were analyzed using a FACS flow cytometer (BD) followed by FACS Diva software. The F-actin content obtained was normalized calculating the ratio between each stimulated cells-mean fluorescence intensity (MFI) values and the non-stimulated cells MFI from the basal levels of the control cells and expressed as fold change.

### Flow Cytometry

Expression of CXCR4 was evaluated by FACS analysis. Briefly, 1 × 10^5^ cells were collected, washed with PBS, incubated with 5 μg/mL anti-CXCR4 (PercyP-Cy5 anti-human CD184 (CXCR4) Antibody) for 20 min, at room temperature, in the dark, and then resuspended in 1% paraformaldehyde. For intracellular staining, the cells were fixed with 4% paraformaldehyde (10 min, room temperature), permeabilized with permeabilizing solution, containing 0.2% BSA, 0.1% azide, 0.5% saponin dissolved in PBS, and then labeled and resuspended as described above. Fluorescence cell analysis was performed with a FACSCalibur (Becton–Dickinson).

### Immunofluorescence Microscopy

Confocal imaging was carried out using primary antibodies against F-actin (MA1-80729, Invitrogen) and CXCR4 (ab2074, Abcam), and secondary antibodies Alexafluor 488-conjugated anti-mouse (Invitrogen) and Alexafluor 555-conjugated anti-rabbit (Invitrogen). Cells were immobilized on cover slips previously treated with poly-L-lysine (1 mg/mL), fixed with 4% paraformaldehyde for 15 min and permeabilized in PBS/0.5% Triton-X-100 for 15 min. The cells were then blocked with PBS/3% BSA and then incubated with the primary (overnight, 4°C) and secondary (2 h, room temperature) antibodies. Slides were mounted using the ProLong Gold antifade reagent with DAPI (Molecular Probes) and examined in the National Institute of Science and Technology on Photonics Applied to Cell Biology (INFABIC) at UNICAMP, using a Zeiss LSM 780-NLO confocal on an Axio Observer Z.1 microscope (Carl Zeiss AG). Images were collected using 1,024 × 1,024 image format and 63 × optical zoom. In the absence of primary antibodies, staining of secondary antibodies (negative controls) failed to produce any significant staining.

### Western Blotting

Equal amounts of protein were used for total extracts with specific antibodies followed by electrophoresis on 8–15% SDS polyacrylamide gels under reducing conditions, transferred to nitrocellulose membranes, stained with appropriate primary antibodies and infrared secondary antibodies. The membranes were visualized with chemiluminescence using an ECL Plus (GE-Healthcare). Quantitative analyses of the optical intensities of the protein bands were determined with Un-Scan-It Gel 6.1 (Silk Scientific Inc.). Monoclonal antibodies against P70S6K (sc-8418) and polyclonal antibodies against AKT1/2/3 (sc-8312), GAPDH (sc-32233), p-AKT1/2/3 (sc-33437), and p-P70S6K (sc-7984) were purchased from Santa Cruz Biotechnology. ERK1/2 [(pT185/Y187); 44-680G] and ERK1/2 (44-654G) from Invitrogen and pHCK (Y410; ab61055) and HCK (ab75839) from Abcam.

### Statistical Analysis

Statistical analysis was performed using GraphPad Prism5 software (San Diego, CA, United States). Data were expressed as median (minimum–maximum). For comparisons, an appropriate Anova, Mann–Whitney test or Student’s *t*-test was used. The values of *P* < 0.05 were considered as statistically significant. All experiments were repeated, at least, four independent times.

## Results

### HCK Knockdown Impaired CXCL12-Induced Chemotaxis in Human Myeloid Leukemia Cells

**FIGURE 1:**
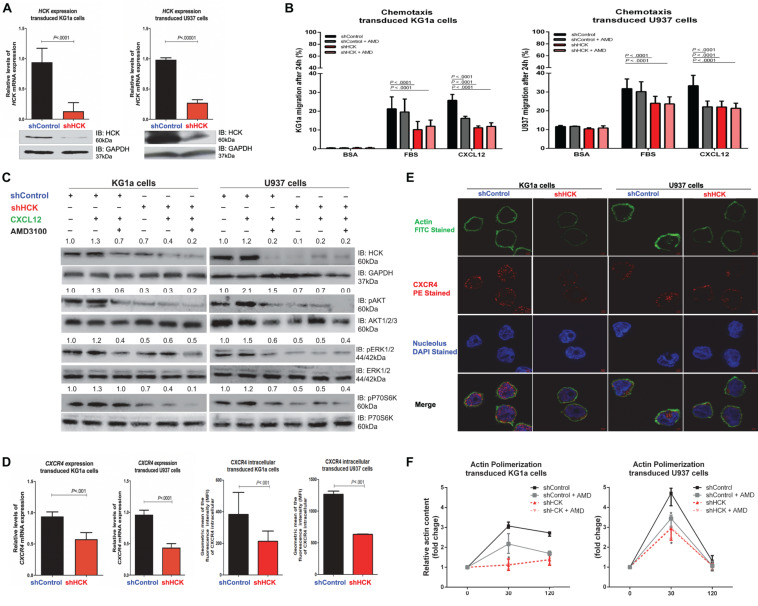
HCK inhibition downregulates CXCR4/PI3K or MAPK pathways in leukemic cell lines, resulting in a reduction of cell chemotaxis through actin polymerization modulation. **(A)** *HCK* mRNA expression and HCK protein levels in transduced leukemia cell extracts. For qRT-PCR, results were calculated for each sample relative to the expression of the endogenous gene and determined using the 2^–ΔΔ*CT*^ method. All data have been normalized to the values of the control. For Western blotting, membranes were blotted with antibodies against HCK (60 kDa) or GAPDH (37 kDa), as a control for equal sample loading, and analyzed by chemiluminescence. **(B)** Chemotaxis assays in transduced leukemia cells. The Transwell system was used to detect the chemotaxis of shControl or shHCK cells toward CXCL12 (100 ng/mL), in the presence and absence of a CXCR4 antagonist (1.25 μg/mL of AMD3100), using 10% FBS-containing medium as a positive control and 0.5% BSA-containing medium as the negative control. After 24 h, the migrated cells on the lower chamber membrane were counted. **(C)** Western blotting analysis of total cell extracts from transduced leukemia cell lines, stimulated or not with CXCL12 (100 ng/mL) and in the presence or absence of a CXCR4 antagonist (AMD3100). Membrane was blotted with antibodies against HCK (60 kDa), pAKT (60 kDa), AKT (60 kDa), pP70S6K (70 kDa), P70S6K (70 kDa), pERK1/2 (44/42 kDa), ERK1/2 (44/42 kDa) or GAPDH (37 kDa), as a control for equal sample loading, and developed with the ECL Western Blotting Analysis System. **(D)** *CXCR4 mRNA* expression and CXCR4 intracellular levels in transduced leukemia cells. For qRT-PCR, results were calculated for each sample relative to the expression of the endogenous reference *HPRT* gene and determined using the 2^–ΔΔ*CT*^ method. For flow cytometry, results were calculated using the MFI of CXCR4 intracellular expression after cell permeabilization. **(E)** Confocal microscopy changes in CXCR4 and F-actin cytoskeleton organization in transduced leukemia cell lines. shControl or shHCK cells were displayed with phalloidin (green), CXCR4 (red), and DAPI (blue) stains by 63 × oil immersion objectives. The black bars/lines represent results obtained from shControl cells, whereas the red bars/lines represent shHCK cells. **(F)** F-actin intracellular levels measured by flow cytometry in transduced leukemia cells. shControl or shHCK cells were stimulated with CXCL12 (300 ng/mL) for 30 or 120 s, in the presence and absence of a CXCR4 antagonist (AMD3100), stained with phalloidin, and then MFI was quantified by flow cytometry. Data indicate fold increase in F-actin content following stimulation with CXCL12. Results are shown as mean ± standard deviation and are representative of, at least, 4 independent experiments; Mann–Whitney test or Student’s one-tailed *t*-test.

In order to investigate the role of HCK on CXCL12/CXCR4 axis in leukemia cells, two human myeloid leukemia cell lineages, the U937 (AML CD34 negative cells) and the KG1a (AML CD34 positive cells) were stably transduced with lentivirus-mediated shRNA targeting *HCK* gene (shHCK) or an appropriate control (shControl). After a polyclonal cell selection with antibiotics, the efficiency of HCK silencing was verified by qPCR and Western blotting (Figure 1A). Our data showed an average *HCK* gene inhibition of 72.0% (range 71.4–72.7%) in KG1a cells and of 73.1% (range 70.1–76.2%) in U937 cells.

Next, we tested whether HCK inhibition was able to modulate CXCL12-induced chemotaxis of leukemia cells using a Transwell-based migration assay. Our data showed that, for both leukemia cell lines, after 24 h without any chemotaxis stimulus (negative control), very few shControl and shHCK cells migrated. On the other hand, the migration capacity of shHCK cells toward a CXCL12 chemokine-driven stimuli was significantly decreased (KG1a cells: average 10.8%, range 10.4–12.3%, and U937 cells: average 22.0%, range 19.2–25.3%) compared to shControl cells (KG1a cells: average 26.8%, range 22.7–29.0%, and U937 cells: average 33.3%, range 27.0–36.8%; *P* < 0.01, Figure 1B). Further, when control cells (shControl) were preincubated with CXCR4 antagonist (AMD3100), prior to the chemotaxis assay, CXCL12 was no longer able to induce leukemia cells migration. Moreover, the migration capacity of shHCK cells toward a CXCL12 chemokine-driven stimuli, in the presence or absence of CXCR4 antagonist (AMD3100), was significantly decreased when compared to shControl cells. Interestingly, in a broadly similar manner, preincubation of the control cells with AMD3100 as well as genetic inhibition of HCK, with or without pretreatment with AMD3100, abrogated the increase in chemotactic migratory ability stimulated by CXCL12 (*P* < 0.01, Figure 1B).

### HCK Silencing Modulated Migration-Associated Pathways in Human Myeloid Leukemia Cells

To further investigate the diminished leukemic cell responsiveness to CXCL12, we evaluated pathways that could be involved in the reduced migration induced by *HCK* gene inhibition. Hence, we attempted to verify pathways overexpressed in hematological malignancies, such as PI3K (Sanchez et al., 2019) and MAPK (Braicu et al., 2019). Thus, we analyzed the phosphorylation levels of AKT, P70S6K and ERK proteins, which were previously demonstrated as downstream of CXCR4 and are usually highly expressed in AML cells. Interestingly, our results showed that the phosphorylation levels of ERK, AKT and P70S6K were decreased in both shHCK KG1a and shHCK U937 cells compared to shControl cells (Figure 1C). Additionally, in shControl transduced cells, CXCL12 stimulus for 5 min resulted in an increase of HCK, AKT, ERK, and P70S6K phosphorylation levels, while combined treatment with CXCL12 and AMD3100 induced an opposite result. Moreover, shHCK transduced cells also showed a significant reduction of HCK, AKT, P70S6K, and ERK phosphorylation compared with shControl cells. However, in shHCK cells, CXCL12 treatment did not modify the phosphorylation of these proteins, suggesting that HCK is a downstream target of CXCR4.

Since *CXCR4* gene expression could be induced by PI3K/AKT signaling (Phillips et al., 2005; Zhou et al., 2015; Chen et al., 2020), we also analyzed *CXCR4* gene expression and CXCR4 intracellular protein content after *HCK* gene inhibition. The qRT-PCR analysis demonstrated a downregulation of *CXCR4* gene expression in shHCK cells compared to shControl cells (average reduction of 38.6% for KG1a cells, and 54.5% for U937 cells; *P* < 0.001) (Figure 1D). Likewise, we found through flow cytometry that CXCR4 intracellular content was reduced in cells with HCK depletion (Figure 1D). The mean fluorescence intensity (MFI) of shHCK cells were 213.63 (range 116.0–330.00) for KG1a cells, and 634.00 (range 626.00–642.00) for U937 cells while shControl cells exhibited 385.25 (range 243.00–658.00) for KG1a cells, and 1266.00 (range 1211.00–1321.00) for U937 cells. These data were confirmed by confocal analysis (Figure 1E) of transduced cells fixed, permeabilized and stained with anti-CXCR4 antibody (red), and with the nuclear stain DAPI (blue). There is no significant difference of surface CXCR4 expression between shControl and shHCK, in both leukemic transduced cells (KG1a and U937) (data not shown).

### HCK Depletion Reduced the Actin Cytoskeleton in Human Myeloid Leukemia Cells

We then evaluated the effect of HCK depletion on actin cytoskeleton dynamics since cell chemotaxis/migration ability depends, in part, of actin polymerization. Leukemia cell lines with *HCK* gene silenced or not, were induced with the CXCL12, a ligand of CXCR4, and then stained with phalloidin, a high-affinity F-actin probe for detection of actin polymerization by flow cytometry analysis. shHCK cells showed a significant reduction of actin polymerization compared to shControl cells. shControl KG1a cells exhibited an expected peak of actin polymerization after 30 s of stimulation with CXCL12 (3.11-fold), mildly reduced after 120 s (2.70-fold) while shHCK KG1a cells showed 1.13-fold increase at 30 s and 1.39-fold increase at 120 s. shControl U937 cells exhibited a high peak at 30 s (4.63-fold) and return to basal levels at 120 s (1.26-fold) while shHCK U937 cells showed 2.96-fold at 30 s and 1.07-fold at 120 s (*P* < 0.01) (Figure 1F). When shControl cells were preincubated with the CXCR4 antagonist (AMD3100), actin polymerization after 30 s of CXCL12 stimulus was 3.39-fold increase in shControl U937 cells and 2.21-fold increase in shControl KG1a cells, which is significantly reduced when compared to shControl U937 cells without AMD3100 addition. Moreover, after preincubation with AMD3100, shHCK cells showed similar response that shHCK cells alone: 3.02 shHCK U937 cells and 1.46 shHCK KG1a cells (Figure 1F). These results were confirmed by immunofluorescence analysis showing that, in both leukemia cell lines, the shControl cells exhibited a higher expression of F-actin compared to shHCK cells (Figure 1E).

### HCK Knockdown Impaired CXCL12-Driven Migration of Leukemic CD34 Positive Cells Isolated From Bone Marrow of AML Patients

**FIGURE 2:**
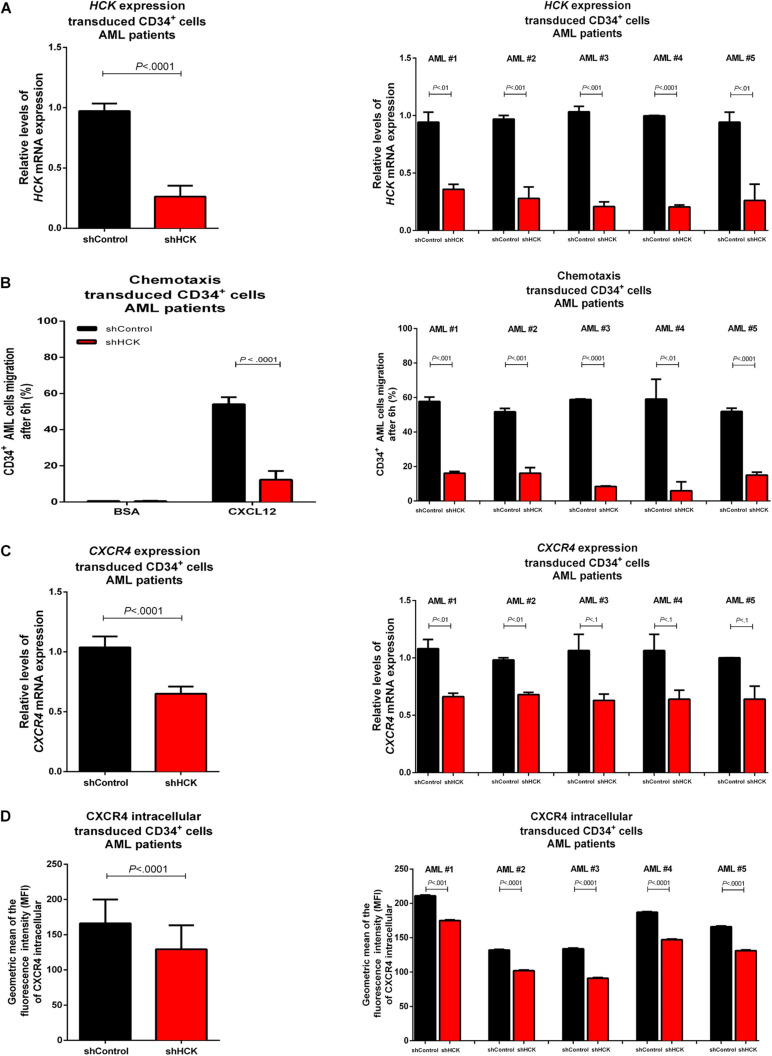
HCK acts on CXCL12 chemotaxis in leukemia stem cells isolated from AML patients bone marrow. **(A)** *HCK mRNA* expression by qRT-PCR in transduced CD34 positive cells. Results were calculated for each sample relative to the expression of the endogenous reference *HPRT* gene and determined using the 2^–ΔΔ*CT*^ method. All data have been normalized to the values of the control. **(B)** Chemotaxis assays in transduced CD34 positive cell. The Transwell system was used to detect the chemotaxis of shControl or shHCK cells toward CXCL12 (100 ng/mL), using 10% FBS-containing medium as a positive control, and 0.5% BSA -containing medium as the negative control. After 6 h, the migrated cells on the lower chamber membrane were counted. **(C)** *CXCR4 mRNA* expression in transduced CD34 positive cell. Results were calculated for each sample relative to the expression of the endogenous reference *HPRT* gene and determined by the 2^–ΔΔ*CT*^ method. **(D)** CXCR4 intracellular levels in transduced CD34 positive cells. For flow cytometry, results were calculated using the MFI of CXCR4 intracellular expression after cell permeabilization. All data are represented as the mean of all 5 patients tested (graphic in the left) and the mean of the individual patient (graphic in the right). Results are shown as mean ± standard deviation and are representative of, at least, 4 independent experiments; Mann–Whitney test.

CD34 positive cells were isolated from bone marrow of AML patients (*n* = 5) collected at diagnosis. The average of bone marrow blasts was 77.88% (range 68–88%, being AML #1: 76%, AML #2: 68%, AML #3: 88%, AML #4: 85%, and AML #5: 72%). AML CD34 positive cells were transduced with lentivirus-mediated shRNA targeting *HCK* gene (shHCK) or an appropriate control (shControl). Efficiency of *HCK* gene silencing, measured by qPCR, showed an average of 71.2% knockdown (range 62.0–80.5%) (Figure 2A). We then evaluated the effects of *HCK* inhibition on CXCL12-driven chemotaxis by Transwell-based migration assay. We observed that, without stimulus, shControl and shHCK cells showed low migration, with no significant differences. However, after CXCL12 induction, shHCK CD34 positive cells obtained from AML patients exhibited lower migration (average: 13.3%; range 5.9–16.1%) compared to shControl CD34 positive cells (average: 53.1%; range 50.9–57.7%; *P* < 0.0001) (Figure 2B). To further investigate the reduced cell responsiveness to CXCL12, we evaluated whether *HCK* knockdown could modulate CXCR4 gene and protein expression. qRT-PCR showed a downregulation in *CXCR4* gene expression of shHCK cells compared to shControl cells (reduction of 36.0%; *P* < 0.0001) (Figure 2C). Flow cytometry analysis of CXCR4 intracellular content revealed diminished levels in shHCK CD34 positive cells (MFI mean: 132; range 102–175) compared to shControl CD34^+^ cells (MFI mean: 166; range 132–211; *P* < 0.001) (Figure 2D).

### HCK Inhibitor (iHCK-37) Reduced Chemotaxis Responsiveness to CXCL12 Stimulus

**FIGURE 3:**
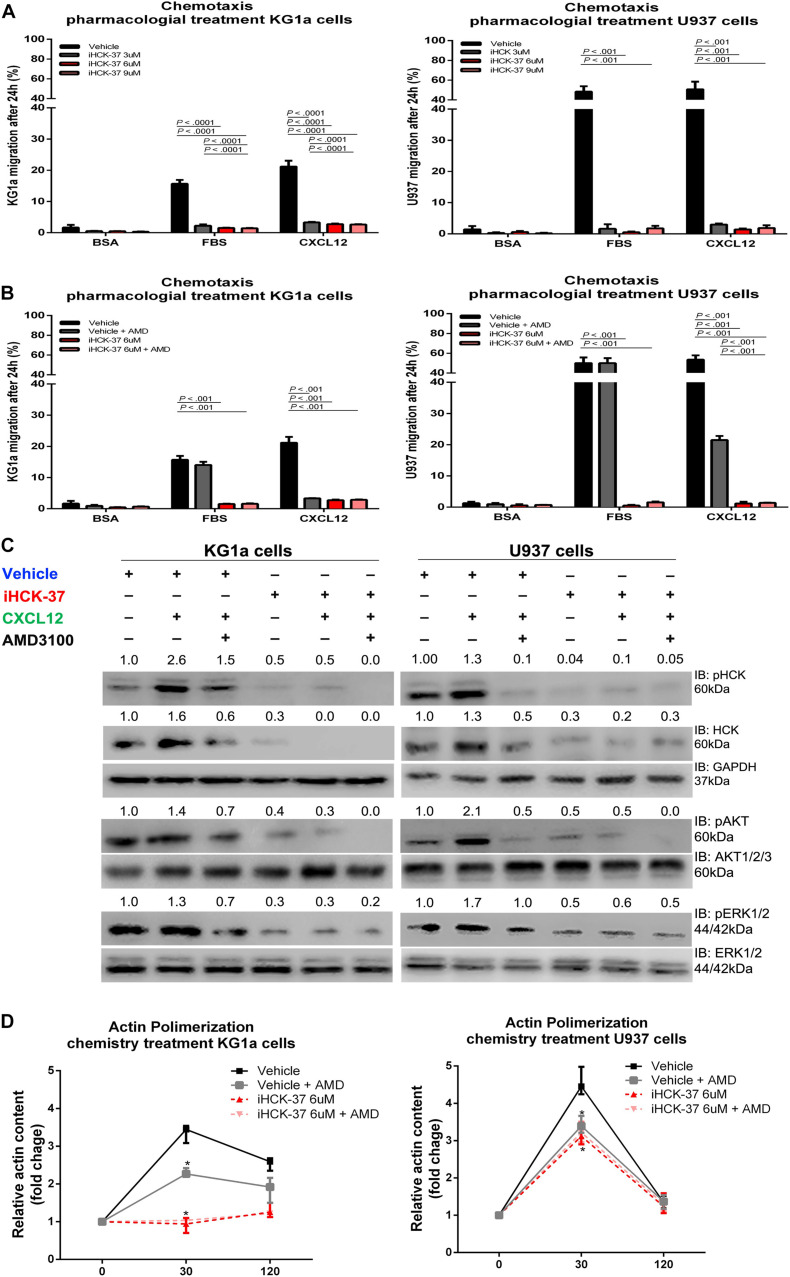
Selective HCK inhibitor reduces CXCR4/PI3K and MAPK signaling pathways activation and decreases cell chemotaxis and actin polymerization. **(A)** Chemotaxis assays in iHCK-37 (3, 6, and 9 μM) treated or not cells and allowed to migrate in Transwell chambers. **(B)** Chemotaxis assays in iHCK-37 (6 μM) treated or not cells, in the presence or absence of CXCR4 antagonist (1.25 μg/mL of AMD3100). Leukemia cell lines were pretreated or not with iHCK-37 for 48 h and allowed to migrate in Transwell chambers toward CXCL12 (100 ng/mL), using 10% FBS-containing media as the positive control and 0.5% BSA-containing medium as the negative control. After 24 h, the migrated cells on the lower chamber membrane were counted. **(C)** Western blotting analysis of total iHCK-37 treated (6 μM) or not cell extracts, stimulated of not with CXCL12 (100 ng/mL) and in the presence and absence of a CXCR4 antagonist (AMD3100). Membranes were blotted with antibodies against HCK (60 kDa), pAKT (60 kDa), AKT (60 kDa), pERK1/2 (44/42 kDa), ERK1/2 (44/42 kDa), or GAPDH (37 kDa), as a control for equal sample loading, and developed with the ECL Western Blotting. **(D)** F-actin intracellular levels measured by flow cytometry in iHCK-37 treated or not cells. Leukemia cell lines were pretreated or not with iHCK-37 (6 μM) for 48 h and then stimulated with CXCL12 (300 ng/mL) for 30 or 120 s, in the presence and absence of a CXCR4 antagonist (AMD3100), stained with phalloidin, and MFI was quantified by flow cytometry. Data indicate fold increase in F-actin content following stimulation with CXCL12. Results are shown as mean ± standard deviation of duplicate replicate and are representative of, at least, 4 independent experiments; Mann–Whitney test.

To confirm the involvement of HCK in CXCL12/CXCR4 axis, we tested a new selective HCK inhibitor, called iHCK-37, synthesized by Dr. Maurizio Botta (*in memoriam*) (Tintori et al., 2013). The inhibition of 50% growth (GI_50_) of KG1a and U937 cells, induced by iHCK-37, was previously determined by us (Roversi et al., 2017). KG1a and U937 cells were pretreated with different doses of iHCK-37 for 48 h (3, 6, and 9 μM corresponding, respectively, to half of GI50, to GI50 and to two times GI50) and then subjected to Transwell-based migration assays in the presence or absence of a stimulus, CXCL12, for an additional 24 h. The iHCK-37 pretreatment in the three tested doses significantly reduced CXCL12–induced cell chemotaxis (KG1a cells: average 2.9%, range 2.5–3.5%, and U937 cells: average 2.0%, range 0.5–3.6%) compared to vehicle treated cells (KG1a cells: average 21.2%, range 19.4–23.3%, and U937 cells: average 33.3%, range 50.8–57.5%; *P* < 0.01, Figure 3A). However, CXCL12 was no longer able to induce migration of cells treated with vehicle and AMD3100 or with iHCK-37 (6 μM) (Figure 3B). Thus, the use of the HCK chemical inhibitor reproduced the results obtained with *HCK* lentivirus inhibition and demonstrated that HCK performs an important role in the migration of leukemic cells probably through CXCL12/CXC4 signaling.

### HCK Inhibitor (iHCK-37) Reduced Activation of PI3K and MAPK Pathways

For a deeper analysis of the molecular and signaling modulation induced by iHCK-37 in leukemic cells, we investigated migration-associated pathways, specifically PI3K and MAPK. Upon pretreatment with iHCK-37 (6 μM) for 48 h, we observed a reduction in activation and expression of HCK, indicating its specificity (Figure 3C). Our data further showed that, in both KG1a and U937 cells, iHCK-37 reduced the phosphorylation of AKT and ERK compared to vehicle treated cells (Figure 3C). Besides, after CXCL12 stimulus, no modulation of phosphorylation levels was observed in cells pretreated with iHCK-37 (6 μM). Further, in cells treated with vehicle, CXCL12 stimulus for 5 min raised the phosphorylation levels of HCK, AKT, ERK, and P70S6K. However, CXCL12 no longer induced phosphorylation of these proteins in cells treated with the CXCR4 antagonist (AMD3100). Thus, the selective iHCK-37 inhibitor was able to reduce HCK protein levels and modulate PI3K and MAPK signaling. All these results corroborated those observed in sh*HCK* cells.

### Actin Polymerization Was Decreased in Leukemic Cells Treated by HCK Inhibitor (iHCK-37)

Therefore, we performed a flow cytometry analysis to evaluate whether iHCK-37 pretreatment was also able to disturb actin polymerization as occurred after *HCK* gene depletion by lentivirus. Leukemia cell lines were pretreated or not with iHCK-37 (6 μM) for 48 h and stained with anti-phalloidin antibody for detection of actin polymerization by flow cytometry. In both leukemic cell lines, cells that were treated with vehicle resulted in a more prominent conversion of globular into F-actin at 30 s after addition of the CXCL12 stimulus (3.63-fold for KG1a cells, and 4.52-fold for U937 cells) (Figure 3D). Notably, the vehicle pretreated KG1a cells maintained a higher amount of F-actin, even 120 s after CXCL12 stimulation (2.56-fold), which was not observed in the vehicle pretreated U937 cells (1.37-fold). Moreover, pretreatment of both leukemic cells with iHCK-37 resulted in a significant reduction of actin polymerization (KG1a cells: 0.95-fold, and U937 cells: 3.12-fold) compared to untreated cells (KG1a cells: 3.36-fold, and U937 cells: 4.52-fold) after 30 s of CXCL12 induction (*P* < 0.001) (Figure 3D), confirming the results obtained with cells *HCK* inhibited by lentivirus. Besides, a significant reduction of actin polymerization was verified in vehicle treated cells incubated with AMD3100 as wells as in iHCK-37 (6 μM) treated cells, in the presence or absence of AMD31000, when compared to vehicle treated cells without AMD3100.

## Discussion

The crosstalk between leukemic stem/progenitor cells and bone marrow microenvironment has been highlighted as an important process for the resistance to chemotherapy and disease relapse in AML, mainly through the activation of the CXCL12/CXCR4 pathway (Bernasconi and Borsani, 2019).

CXCL12, a chemokine secreted by BM mesenchymal stromal cells, binds and triggers its receptor, the CXCR4, on normal or leukemic hematopoietic cells, activating multiple signaling pathways (Shafat et al., 2017), such as PI3K and MAPK (Cojoc et al., 2013), through different intermediate molecules, as Src kinase family (Amata et al., 2014). This activation results in the modulation of various biological processes, such as chemotaxis, adhesion, migration, cell proliferation, and survival (Amata et al., 2014; Zhou et al., 2019). Interestingly, leukemic stem/progenitor cells are more effective in using the crosstalk with their neighboring BM-MSC and BM extracellular matrix to create specific reciprocal dependency in which microenvironment constitutive generate growth-promoting and anti-apoptotic signals for their maintenance (Wang and Zhong, 2018). Thus, leukemia cell migration and retention in the bone marrow microenvironment are important processes in cancer progression as LSC becomes resistant to chemotherapy-induced apoptosis and improves immune escape by limiting antileukemic T-cell responses (Riether et al., 2015; Siveen et al., 2017). In this way, CXCL12/CXCR4 signaling could represent a potential therapeutic target for AML and compounds that acts blocking CXCR4 activation could be useful.

Previously we observed overexpression of HCK, a SFK member expressed in myeloid cells (Dos Santos et al., 2008), in primary CD34 positive cells and total bone marrow (CD34^+^/CD38^+^) cells isolated from AML and MDS patients when compared with healthy donor cells. Interestingly, HCK was very low expressed in mesenchymal stromal cells (CD34^–^/CD38^–^). Moreover, we demonstrated that HCK probably is an upstream regulator of AKT and ERK (Baratti et al., 2010; Roversi et al., 2017). As Src kinase family was described as a player in CXCL12/CXCR4 and PI3K or MAPK pathway in severe solid tumor, we hypothesized whether HCK could be a downstream target of CXCL12/CXCR4 pathway and contribute to the pathophysiology of leukemia cells. In this wise, to elucidate the possible role of HCK in CXCL12/CXCR4 axis, we used a lentiviral vector delivering shRNA specific to the human *HCK* gene as well as a specific pharmacological inhibitor (iHCK-37) to reduce HCK protein levels in two well-established models of myeloid cell lineages, KG1a and U937 cells. KG1a is a CD34 positive AML cell line described to be stem-like while U937 is a pro-monocytic human myeloid leukemia cell line (She et al., 2012). As one major problem of using shRNAs in experimentation is the possibility of off-target effects, a pool of 3 shRNA (Santa Cruz Biotechnology) and a construction of shRNA-GFP vector were used to enable the discrimination of knockdown and general effects of transfection, including toxicity due to spinoculation, liposome or antibiotic selection.

To examine the possible role of HCK on the CXCL12/CXCR4 pathway, we first performed a Transwell chemotaxis assays, using CXCL12 as a chemotactic stimulus to mimic the CXCL12 secretion by BM mesenchymal stromal cells (Dar et al., 2005). After a period to allow for migration, our results showed that leukemic cell lineages with HCK reduced gene and protein expression had significantly decreased capacity of migrating through chemokine-driven stimuli compared to control transduced cells or cells treated with vehicle. Interestingly, the lower ability of chemotaxis after HCK depletion is similar to those observed after control cells (transduced or treated with vehicle) were incubated with AMD31000, a highly specific chemokine CXCR4 antagonist (DiPersio et al., 2009). As cell motility requires a cytoskeleton rearrangement and actin polymerization, we also examined the effect of HCK inhibition on CXCL12-induced activation of the motility machinery. We observed that control or cells treated with vehicle exhibited an expected peak of actin polymerization after a brief stimulation with CXCL12 (approximately 30 s), which, as expected, was no more observed after incubation with AMD31000. On the other hand, HCK gene and protein depletion, in presence or absence of AMD3100, caused a reduction of action polymerization in human myeloid leukemia cell lines compared to control or cells treated with vehicle. Thereby, HCK probably act upon the migration of leukemic cells through CXCL12/CXCR4 pathway, resulting in an increased homing potential to the protective bone marrow microenvironment. Thus, we could hypothesize that the depletion of HCK is able to reduce the chemotaxis of the leukemic cells to the bone marrow niche, avoiding their survival and protection from chemotherapy.

Next, we investigated some possible HCK downstream targets that could contribute to chemotaxis and actin polymerization. As SFK can act on several pathways, we chose to analyze pathways that are abnormally activated in leukemia cells and involved in cell motility, migration and chemotaxis, and that is described as in CXCL12/CXCR4 signaling (Zhou et al., 2015). Thus, we investigated whether the activation of AKT/P70S6K, and ERK, which are essential proteins of the PI3K (Sanchez et al., 2019) and MAPK (Braicu et al., 2019) pathways, respectively, is dependent on HCK. Following a short exposure to CXCL12, control cells showed an increase in phosphorylation levels of AKT and ERK while pretreatment with the CXCR4 antagonist (AMD31000) partially prevented CXCL12-induced phosphorylation. Interestingly, HCK depletion by lentivirus or by specific pharmacological inhibitor decreased the phosphorylation levels of AKT, P70S6K, and ERK1/2, even after a short CXCL12 stimulation. These results suggest that HCK might be an intermediate target of CXCL12/CXCR4 and PI3K or MAPK pathway. Interestingly, we observed a downregulation in *CXCR4* gene and CXCR4 intracellular protein expressions after HCK depletion of leukemic cells. These findings could be related to the role of phospho-P70S6K to regulate the expression of *CXCR4* (Phillips et al., 2005; Zhou et al., 2015; Chen et al., 2020).

**FIGURE 4:**
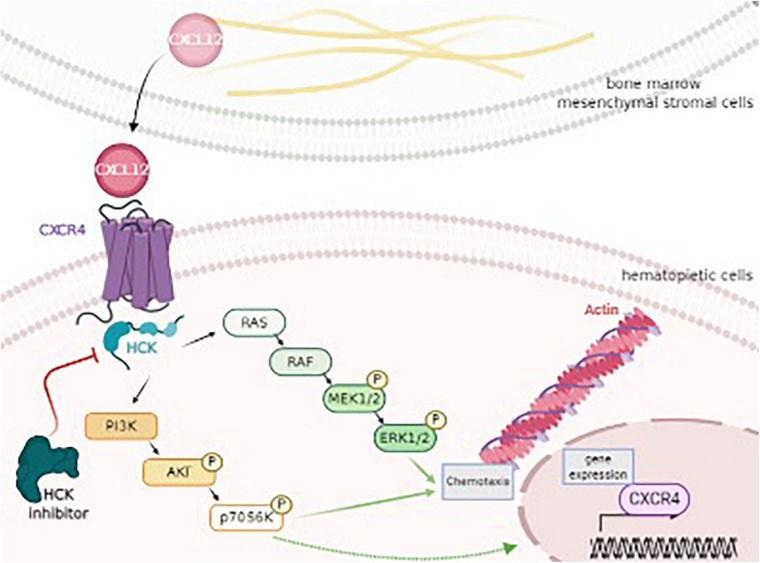
HCK as a key target of CXCL12, CXCR4, PI3K/AKT, and MAPK/ERK signaling pathway, modulating chemotaxis and cytoskeleton activity. CXCL12 is secreted by bone marrow mesenchymal stromal cells and binds and triggers its receptor, CXCR4 on hematopoietic cells. This interaction results in the activation of HCK and, consequently, PI3K/AKT/P70S6K and MAPK/ERK pathways, regulating processes as chemotaxis, cytoskeleton (actin polymerization) and gene expression (*CXCR4*). Thus, HCK could control the leukemic cell homing to the protective BM niche by a migratory advantage of leukemic cells and could represent a potential target for new therapeutical approaches for AML. Figure was produced using Servier Medical Art. http://www.servier.com/Smart/ImageBank.aspx?id=729.

In conclusion, to the best of our knowledge, the present study was the first to demonstrate that HCK could be an intermediate target of CXCR4 and PI3K or MAPK pathways, resulting in the modulation of two important biological processes related to leukemic progression (chemotaxis and actin polymerization) (Figure 4). Our study may further the understanding of the role and molecular mechanisms underlying HCK as well as provide new insight into HCK as a potential target for new therapeutic approaches for AML. Pre-clinical studies in AML models and analysis of the association of iHCK-37 with drugs used for AML treatment are desired for translation to the bedside of the results here presented.

## Data Availability Statement

The raw data supporting the conclusions of this article will be made available by the authors, without undue reservation.

## Ethics Statement

The studies involving human participants were reviewed and approved by the Human Ethics Committee of the University. The patients/participants provided their written informed consent to participate in this study.

## Author Contributions

FR was the principal investigator of this study, designed and performed the experiments, collected, analyzed, and interpreted the data, and wrote the manuscript. FP acted as the reference physician and helped analyze the data. MB helped analyze data and wrote the manuscript. SO provided the study conception, directed the research, provided financial support, and revised and gave final approval of the manuscript. All authors have read, commented, and approved the final version of the manuscript.

## Conflict of Interest

The authors declare that the research was conducted in the absence of any commercial or financial relationships that could be construed as a potential conflict of interest.
