# Supplementary figures and images for: Hematopoietic Cell Kinase (HCK) Is a Player of the Crosstalk Between Hematopoietic Cells and Bone Marrow Niche Through CXCL12/CXCR4 Axis
Source: Front Cell Dev Biol. 2021 Mar 25;9:634044. doi: 10.3389/fcell.2021.634044 (PMC8027121; doi:10.3389/fcell.2021.634044)

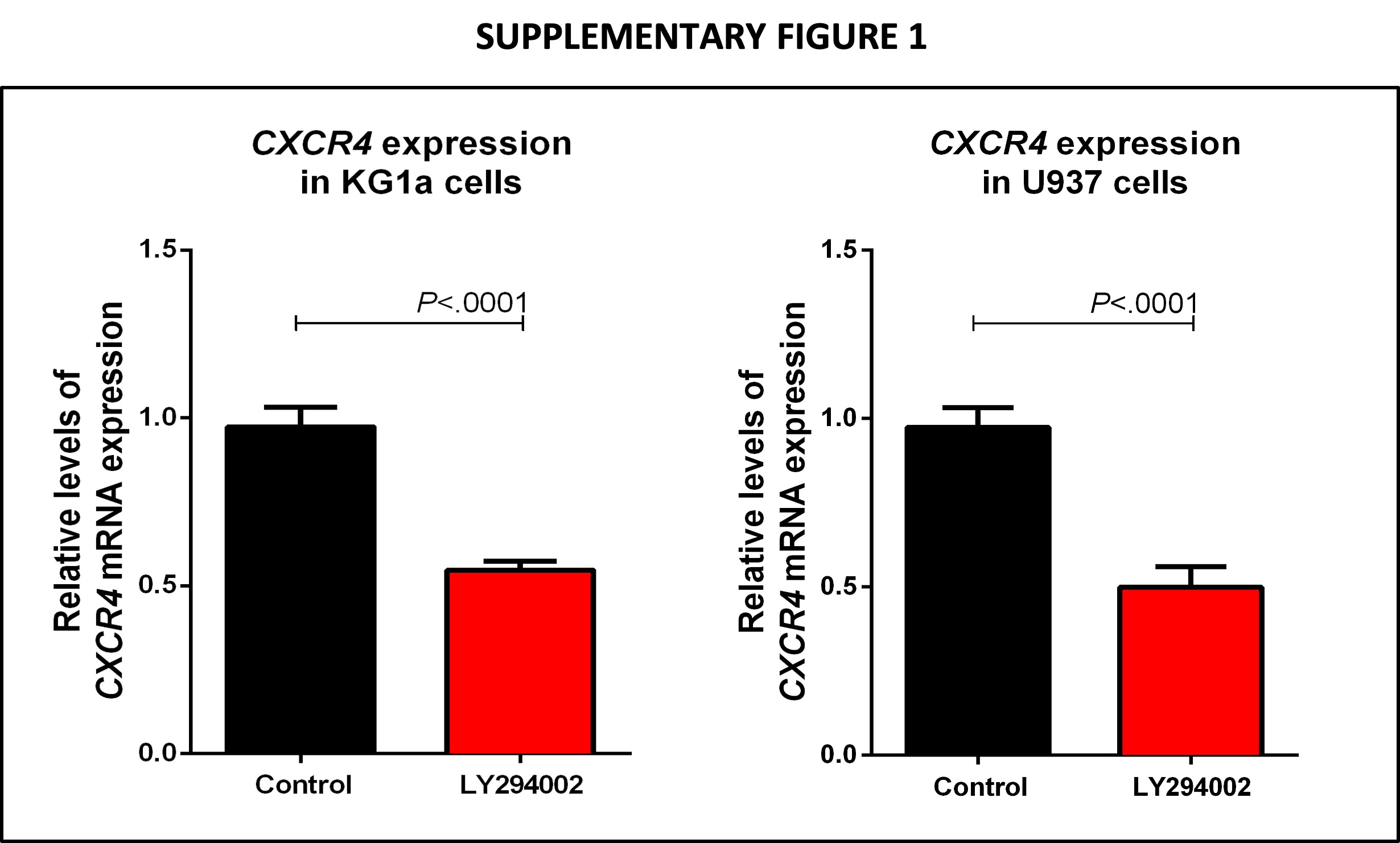

Supplement: Supplementary file 1 [file Image_1.JPEG]
